# A NOTCH added to metabolomics

**DOI:** 10.1038/s41416-019-0463-0

**Published:** 2019-05-22

**Authors:** Odd Terje Brustugun

**Affiliations:** Section of Oncology, Drammen Hospital, Vestre Viken Health Trust, Dronninggata 28, N-3004 Drammen, Norway

**Keywords:** Non-small-cell lung cancer, Mechanisms of disease

## Abstract

Deregulated metabolism is a hallmark of cancer. In the accompanying study by Sellers et al. published in the *British Journal of Cancer*, metabolism-related transcriptomics data from in silico data sets are analysed, with the findings being further investigated in the experiments on tumour tissue slices and finally validated in patients. The study adds to our growing understanding of therapeutically accessible metabolic reprogramming in malignancies.

## Background

Cancer is a heterogeneous group of diseases that are handled according to their biology. Some cancers follow a chronic trajectory, whereas others are extremely aggressive. Lung cancer is in general the deadliest of cancers, but it also is acknowledged as a heterogenous mixture of entities with diverse biological and clinical features.

Non-small-cell lung cancer is subdivided into adenocarcinoma (AC) and squamous cell carcinoma (SCC). The current categorisation based on morphology and a few cell-surface markers detected by immunohistochemistry has proven robust and clinically useful. However, when doing cluster analyses based on a variety of clinical and biological features (“omics”, see below), it is clear that more detailed partitioning is relevant.

In the accompanying paper by Sellers et al.,^1^ a comprehensive analysis of metabolomics from in silico to in vivo data sets is presented. This and other studies demonstrate that metabolic reprogramming in tumours is central to the behaviour of cancers, and that understanding the intricate phenomena in tumour metabolism may uncover novel targets for therapy.

## Omics in cancer research

Over the past couple of decades, a multitude of “omics”, such as genomics, epigenomics, transcriptomics, proteomics and, lately, also metabolomics, have provided a wealth of knowledge leading to a deep mechanistic understanding of the information flow from DNA to (patho-)physiology.^2^ At present, the integration of these large data sets is moving further, including semi-external factors such as microbiomics, as well as indirect biological data such as information from imaging and wearable sensors. Integration of all these data sets in routine clinical practice seems unfeasible at the moment due to the costs, computing and storage challenges involved, as well as bioinformatics and interpretation issues. Nonetheless, it is imperative to capture this complexity to understand the biology and the disease. Thus, comprehensive analyses of multiple orthogonal omics profiles of large patient cohorts are needed in research. Hopefully, with time, methods feasible at the individual patient level will emerge, enabling personalised medicine at an extraordinarily detailed molecular granularity. Metabolomics, due to its inherent effect on tumour growth, will probably play a more central role in clinical treatment decisions in the future. Notably, studies in lung cancer have found that increased glucose uptake and activation of glycolysis (i.e. the Warburg effect) lead to altered nucleotide metabolism, which in turn may indicate an opportunity for therapeutic perturbations.^3^ Furthermore, metabolomics studies can benefit from advances within imaging, especially PET-based methods.^4^ Likewise, metabolomics data are already being used in radiotherapy planning, as the dosage of radiation is made dependent on the metabolic features obtained from PET-FDG images.^5^

## NOTCH

Lung SCC remains an entity of unmet medical need. Hitherto, no druggable genetic aberrations have been found in SCC, in contrast with AC, for which several targeted therapies are approved. The finding by Sellers et al.^1^ that SCC may have specific metabolically-reprogrammed pathways that are amenable to therapeutic interventions might clinically be highly relevant. Specifically, the observation of upregulation of Notch1 mRNA expression and subsequent effects on downstream targets in SCCs point to potential druggable targets, in line with other studies.^6^ Genes encoding proteins involved in glucose uptake, glycolysis, lactate to pyruvate conversion and repression of the tricarboxylic acid cycle are direct transcriptional targets of NOTCH signalling. Drugs targeting NOTCH pathways are already in clinical trials, but patient selection based on relevant biomarkers is crucial.^7^ Although our biological understanding of the role of NOTCH currently is limited, the pathway seems to be especially relevant in regulating interactions within the tumour microenvironment, and combination with other therapeutic strategies seems promising.^8^ The in-depth context-based analysis herein,^1^ in contrast with other negative studies on protein expression evaluated by immunohistochemistry, again underscores the importance of comprehensive approaches for uncovering treatable features.

## Research in situ

Cancers are notoriously heterogenous, with mosaics of tumour and normal cell composition both within primary tumours and in and between metastases. In addition, blood perfusion and metabolic heterogeneity are evident in lung tumours.^9^ It is thus clear that using monoclonal cell cultures in cancer research is a very simplistic and artificial approach, and that findings from such systems may lead to erroneous or irrelevant conclusions. Not only are metabolic differences lost in cell culture systems, but also a multitude of tumour/stroma interactions are increasingly recognised as important factors in carcinogenesis. Not the least, the advent of immunotherapy, employing the host’s own T-cells, underscores the shortcomings of cancer studies on cell lines.

In the present study,^1^ many metabolic aspects were lost in the in vitro systems when compared with the in vivo results. Xenografts may indicate more realistically the natural behaviour of a tumour, but studies on human tissue in situ should be the ultimate goal. However, the acquisition process, as well as the interventional obstacles, are obvious challenges. Therefore, the study by Sellers et al.^1^ is a good example of the research process, from in silico studies via in vitro experiments to confirmatory studies in vivo.

For the in vivo studies, Sellers et al.^1^ employed stable isotope-resolved metabolomics (SIRM) to provide a higher level of mechanistic evidence. Epigenetic aberrations and post-translational modifications, as well as temporal and spatial dynamics, may not be detected even via sophisticated methods such as RNA sequencing. Direct measurements of metabolic pathway activation in situ via SIRM may therefore be of great clinical relevance.^10^

## Outlook

Further studies on the metabolomics related to the effects of various therapies are highly awaited; especially interesting is the link to immunotherapy responses. Metabolic features could be relevant in the search for better biomarkers for response prediction – beyond PD-L1 expression. On that note, it is interesting that host metabolism may play a more central role than hitherto believed, similar to how obesity has been found, somewhat surprisingly, to be a positive predictor of response to immunotherapies.^11^

The integrative comprehension of various orthogonal omics provides biological information at an unprecedented detailed level. Factors involving the host, including tumour microenvironment, are increasing the complexity of cancer biology, but understanding the tumour/host interplay is crucial in order to be able to move cancer care into the truly personalised era. Immunotherapy is an example of a treatment where a large fraction of patients do not respond, and improvement in predictive biomarkers is necassary to reduce this. Further research in omics, not the least into metabolomics, will probably lead to improved biomarkers and better treatment outcomes; for example, when combining activation of the host’s immune system using checkpoint inhibitors and perturbation of individual tumour features, as with drugs interfering with the NOTCH pathway.

## Data Availability

Not applicable.
